# Paired genomic profiling of primary tumor and lymph-node metastases identifies candidate prognostic features in penile squamous cell carcinoma

**DOI:** 10.1093/oncolo/oyag319

**Published:** 2026-08-27

**Authors:** Kexin Lou, Linghui Liang, Hongzhou Cai, Ting Xu, Fufeng Wang, Di Wang, Qifan Jing, Jiani C Yin, Xiaoxiang Zhang, Zicheng Xu

**Affiliations:** Department of Pathology, Jiangsu Cancer Hospital & Jiangsu Institute of Cancer Research & Affiliated Cancer Hospital of Nanjing Medical University, Nanjing, 210009, China; Department of Urologic Surgery, Jiangsu Cancer Hospital & Jiangsu Institute of Cancer Research & Affiliated Cancer Hospital of Nanjing Medical University, Nanjing, 210009, China; Department of Urologic Surgery, Jiangsu Cancer Hospital & Jiangsu Institute of Cancer Research & Affiliated Cancer Hospital of Nanjing Medical University, Nanjing, 210009, China; Department of Urologic Surgery, Jiangsu Cancer Hospital & Jiangsu Institute of Cancer Research & Affiliated Cancer Hospital of Nanjing Medical University, Nanjing, 210009, China; Nanjing Geneseeq Technology Inc., Nanjing, 210000, China; Nanjing Geneseeq Technology Inc., Nanjing, 210000, China; Nanjing Geneseeq Technology Inc., Nanjing, 210000, China; Nanjing Geneseeq Technology Inc., Nanjing, 210000, China; Department of Urologic Surgery, The Second People's Hospital of Kunshan, Kunshan, 215300, China; Department of Urologic Surgery, Jiangsu Cancer Hospital & Jiangsu Institute of Cancer Research & Affiliated Cancer Hospital of Nanjing Medical University, Nanjing, 210009, China

**Keywords:** penile squamous cell carcinoma, next-generation sequencing, CCND1/FGF19 co-amplification, tumor mutation burden, prognostic biomarkers

## Abstract

**Background:**

Penile squamous cell carcinoma (PSCC) is a rare malignancy with limited genomic data in Asian populations. Lymph node metastasis heavily dictates prognosis, yet molecular determinants of progression remain poorly understood. We aimed to characterize the genomic landscape and explore candidate prognostic genomic features using paired primary and metastatic PSCC tumors.

**Patients and Methods:**

Targeted next-generation sequencing (437 cancer-related genes) was performed on primary tumors and matched lymph node metastases from 20 Chinese patients. Somatic alterations, intralesional heterogeneity, and tumor mutation burden (TMB) were analyzed and correlated with disease-free survival (DFS) and overall survival (OS).

**Results:**

The most frequent primary tumor mutations included *TP53* (45%) and *TERT* (40%). Notably, *CCND1/FGF19* co-amplification (20% of cases) was associated with inferior DFS (*P* = .027) and showed a trend toward shorter OS (*P* = .050). Conversely, T-cell receptor (TCR) pathway alterations correlated with markedly improved survival. Comparing paired lesions revealed 59.8% shared alterations. Elevated TMB in metastases relative to matched primary tumors was significantly associated with poorer DFS (*P* = .008), while higher intralesional heterogeneity showed a trend toward worse OS.

**Conclusion:**

Paired profiling revealed broadly conserved genomic features together with lesion-specific divergence in PSCC. Recurrent *CCND1/FGF19*-containing 11q13 amplification, TCR pathway alterations, and elevated metastatic TMB warrant evaluation as potential prognostic features in larger, independently validated cohorts with integrated HPV and immune profiling.

Implications for Practice This study adds paired primary-metastatic genomic data from an underrepresented Chinese PSCC cohort. *CCND1/FGF19*-containing 11q13 amplification and elevated metastatic TMB were associated with adverse outcomes, whereas TCR pathway alterations were associated with favorable survival. Although these findings require validation in larger independent cohorts, they suggest that paired primary-metastatic genomic profiling may help identify candidate features for future postoperative risk stratification. These results also provide a rationale for future translational studies evaluating the biological and therapeutic relevance of 11q13 amplification, metastatic TMB dynamics, and immune-related genomic alterations in PSCC.

## Introduction

Penile cancer (PC) is a rare malignancy with significant geographical variations in incidence. It is relatively uncommon in developed countries but occurs more frequently in developing regions, where it poses a substantial public health burden.^1^ According to the National Central Cancer Registry of China, the age-standardized incidence of PC among Chinese men in 2011 was 0.60 per 100 000, with a mortality rate of 0.18 per 100 000.^2^ Despite its rarity, PC has considerable clinical impact due to its high risk of local invasion and lymphatic metastasis, particularly when diagnosis is delayed.^3^

The predominant histological subtype of PC is squamous cell carcinoma (SCC), accounting for more than 95% of cases.^4^ Known risk factors include human papillomavirus (HPV) infection, phimosis, poor genital hygiene, smoking, chronic inflammation, and low rates of circumcision.^5^ In China, where circumcision is not routinely performed, many patients present with phimosis or redundant prepuce,^3^ which may contribute to the regional epidemiological patterns of PC.

Lymph node metastasis (LNM) remains the most important prognostic factor in PC. While physical examinations and conventional imaging, including ultrasonography, computed tomography (CT), and magnetic resonance imaging (MRI) are commonly used for nodal assessment, their sensitivity for detecting micrometastasis is limited.^6^^,^^7^ Even sentinel lymph node biopsy, which improves detection accuracy, can miss subclinical disease.^8^ Molecular biomarkers are therefore needed to improve risk stratification and postoperative management.

Several alterations involving *TP53, CDKN2A, PIK3CA, HRAS,* and *STK11* have been reported in PC.^9–13^ However, most studies have involved Western populations.^10^ Genetic studies based on Asian or Chinese cohorts remain limited, and the influence of ethnicity on molecular heterogeneity remains poorly understood. Moreover, few investigations have compared the genomic profiles of primary tumors and matched lymph node metastases, and their relevance to clinical outcomes remains unclear.

In this study, we performed targeted next-generation sequencing (NGS) on both primary tumors and paired lymph node metastases from 20 Chinese patients with penile squamous cell carcinoma (PSCC). We aimed to characterize the somatic mutational landscape in this underrepresented population, identify genomic alterations associated with recurrence and survival, and explore potential molecular differences between primary and metastatic lesions. These findings may contribute to a better understanding of the molecular features of PC and provide preliminary insights into genomic features associated with clinical outcomes and generate hypotheses for future biomarker validation.

## Methods

### Subject selection

This retrospective study included patients with histologically confirmed PSCC who underwent surgery at Jiangsu Province Cancer Hospital from April 2019 to October 2024. The study protocol was approved by the Ethics Committee of Jiangsu Province Cancer Hospital (Approval No. KY-2025-050), and all participants provided written informed consent prior to enrollment.

The inclusion criteria were as follows: (1) male patients diagnosed with PSCC who underwent definitive surgery with or without inguinal lymph node dissection; (2) availability of sufficient formalin-fixed paraffin-embedded (FFPE) tumor tissue for genomic testing; (3) no history of prior systemic or local antitumor therapy before surgery; (4) age ≥18 years at diagnosis; and (5) availability of complete clinical and follow-up data. Exclusion criteria included: (1) history of other malignancies; (2) inadequate tumor content for sequencing; or (3) refusal to participate in the study. Patients with distant metastasis were included only when all known metastatic lesions were subsequently completely resected and/or treated with curative intent and no residual disease was documented after completion of treatment.

### Sample collection and preparation

Primary tumor samples were available from all 20 patients included in the study. Paired LNM samples were available from 18 of these patients during surgical procedures. All tumor tissues were FFPE and were reviewed by two independent pathologists to confirm tumor content. For NGS, samples with a minimum of 20% tumor cellularity were included to ensure adequate DNA quantity for analysis.

### DNA extraction and sequencing library construction

FFPE tumor tissues were subjected to targeted NGS using a comprehensive 437-gene solid tumor panel (GeneseeqPrime™, Geneseeq Technology Inc.) at a Clinical Laboratory Improvement Amendments (CLIA)-certified and College of American Pathologists (CAP)-accredited laboratory (Nanjing Geneseeq Technology Inc., Nanjing, China). DNA was extracted using the QIAamp DNA FFPE Tissue Kit (Qiagen), followed by library construction with the KAPA Hyper Prep Kit (KAPA Biosystems) according to standard protocols.^14^^,^^15^

Target enrichment was performed using custom xGen Lockdown Probes (Integrated DNA Technologies), designed to capture 437 cancer-related genes. Hybridization was carried out with Dynabeads M-270 (Life Technologies). The prepared libraries were amplified with KAPA HiFi HotStart ReadyMix using Illumina p5 (5′-AAT GAT ACG GCG ACC GA-3′) and p7 primers (5′-CAA GCA GAA GAC GGC ATA CGA GAT-3′), and purified using Agencourt AMPure XP beads. Library concentration and quality were assessed using the KAPA Library Quantification Kit and Agilent Bioanalyzer 2100, respectively.^16^^,^^17^ Sequencing was conducted on an Illumina HiSeq4000 platform following the manufacturer’s protocols.

### Mutation calling

Raw sequencing data were demultiplexed using bcl2fastq (v2.16.0.10), and adapter sequences were removed with Trimmomatic.^17^^,^^18^ Reads were aligned to the human reference genome (hg19, GRCh37) using Burrows-Wheeler Aligner (BWA, v0.7.12).^17^^,^^19^ Resulting binary alignment map (BAM) files were processed with Picard (v1.119) and the Genome Analysis Toolkit (GATK, v3.4-0) for duplicate removal, realignment around indels, and base quality score recalibration.^17^

Single-nucleotide variants (SNVs) and small insertions/deletions (indels) were identified using VarScan2^20^ with a minimum variant allele frequency (VAF) of 1% and a significance threshold of *P* < .05. Variant filtering criteria included: (1) minimum depth of 20; (2) base quality ≥15; (3) ≥ 5 supporting reads; (4) presence on both strands; (5) strand bias <10%; (6) population allele frequency <1% in 1000 Genomes or Exome Aggregation Consortium (ExAC)^21^; and (7) exclusion of sequencing artifacts. Variants were annotated with ANNOVAR and visually inspected with the Integrative Genomics Viewer.

Tumor mutation burden (TMB) was defined as the total number of somatic mutations, including synonymous variants, per megabase of coding regions, while excluding known driver hotspots and gene fusions.^22^^,^^23^ Chromosomal instability (CIN) was quantified as the proportion of genome segments exhibiting a log2 copy number ratio deviation greater than ±0.2, reflecting large-scale copy number changes across the genome.^24^

Intralesional heterogeneity (ITH) was used to evaluate genomic divergence between paired primary and metastatic lesions. ITH was defined as the ratio of the total number of mutations uniquely present in either the primary or metastatic sample to the total number of mutations across both sites.

### Follow-up and outcome assessment

Follow-up was conducted beginning approximately one month after surgical treatment. Patients were evaluated every three months during the first year and every 3-6 months thereafter. Clinical assessments included physical examination, imaging studies (ultrasound, CT, MRI, or PET-CT as appropriate), and routine laboratory tests. The last follow-up was completed in January 2025.

Disease-free survival (DFS) was defined as the time from the date of definitive surgery to the first documented recurrence (local or distant) or death from any cause, whichever occurred first. Overall survival (OS) was defined as the time from diagnosis to death from any cause. Patients without events were censored at the date of the last follow-up. The median follow-up duration was [32.4] months (range: [2.0-68.5] months).

### Statistical analysis

Categorical variables are reported as counts and percentages and continuous variables as medians and ranges. In 18 patients with paired primary and metastatic samples, differences in individual gene-level alteration status between lesions were assessed using the exact McNemar test. Paired continuous genomic measures, including TMB, copy number variation (CNV) scores, CIN, and ITH, were compared using the two-sided Wilcoxon signed-rank test. Co-occurrence and mutual exclusivity were assessed using two-sided Fisher exact tests and Benjamini-Hochberg (BH) false-discovery-rate (FDR) correction. Alterations were mapped to canonical oncogenic pathways described by Sanchez-Vega et al.^25^ and immune-related gene sets from ImmPort database.^26^ A pathway was considered altered when at least one constituent gene was altered. Only pathways altered in at least three patients were analyzed for survival.

DFS and OS were estimated using the Kaplan-Meier method and compared using the two-sided log-rank test. Univariate Cox proportional hazards models were used to estimate hazard ratios (HRs) and 95% confidence intervals (CIs). Gene- and pathway-level log-rank *P* values were BH-adjusted separately for DFS and OS within each analysis family; both *P* and *q* values are reported, with *q* < 0.05 indicating statistical significance after correction. These analyses were considered exploratory regardless of significance. For other analyses, a two-sided *P* value <.05 was considered statistically significant. All statistical analyses were conducted using R software (version 3.5.3).

## Results

### Patient characteristics and clinical outcomes

**Figure 1.**
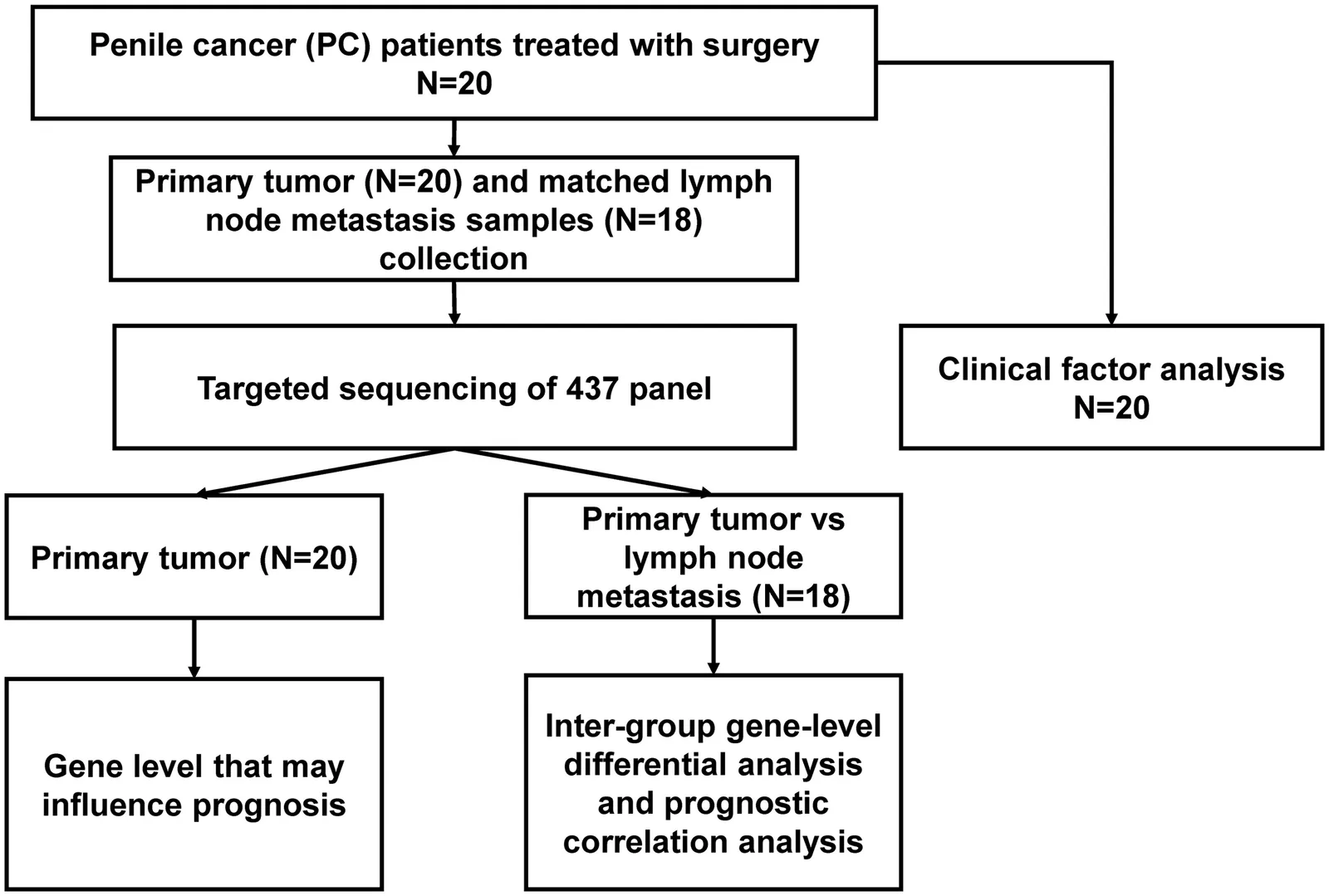
Study design and patient inclusion workflow. A total of 20 penile cancer (PC) patients treated with surgical resection were enrolled. Primary tumor samples (*n* = 20) and matched lymph node metastasis samples (*n* = 18) were collected for targeted sequencing using a 437-gene cancer panel. Clinical data were available for all patients (*n* = 20) and used for prognostic analysis. Genomic profiling of primary tumors aimed to identify potential prognosis-related alterations. Paired analyses of primary and metastatic tumors (*n* = 18) were conducted to evaluate inter-lesion genomic differences and their association with clinical outcomes.

Twenty surgically treated patients with PSCC were included. Primary tumor samples were available from all patients and paired LNM samples were available for 18 of them. The detailed patient inclusion and sample collection process is illustrated in Figure 1. The median age at diagnosis was 58 years (range: 40-92), and all tumors were SCC (Table 1). According to TNM staging, only one patient (5%) was classified as stage II, 12 patients (60%) as stage III (nine IIIA and three IIIB), and seven patients (35%) as stage IV (Table 1). Among the stage IV patients, three had M1 disease, three had M0 disease with N3 lymph node involvement, and one had N3 disease with an indeterminate distant metastasis status (MX). All stage IV patients were treated with curative intent. In the three M1 patients, all known metastatic lesions were completely resected and/or definitively treated, with no documented residual disease after completion of treatment. The patient with MX status underwent curative-intent treatment and had no documented residual disease after treatment, although complete baseline distant-staging documentation was unavailable (Table S3). Regarding histological grading, five patients (25%) were highly-differentiated, nine patients (45%) were moderately differentiated, and five patients (25%) were poorly differentiated. One tumor was classified as intermediate-to-poorly differentiated (Table 1). Sixteen patients (80%) underwent inguinal lymph node dissection at the time of primary tumor surgery, and 12 (60%) received adjuvant therapy (Table 1).

**Table 1.** Clinicopathological characteristics and treatment information of the PC patient cohort (*n* = 20).

Characteristics	*n* (%)
**Age, median (range), year**	58 (40-92)
** <60**	11 (55.0%)
** ≥60**	9 (45.0%)
**TNM stage**	
** II**	1 (5.0%)
** III**	12 (60.0%)
** IV**	7 (35.0%)
**Differentiation**	
** Highly (I)**	5 (25.0%)
** Moderately (II)**	9 (45.0%)
** Moderately-poorly (II-III)**	1 (5.0%)
** Poorly (III)**	5 (25.0%)
**Pathological subtype**	
** SCC**	20 (100.0%)
**Inguinal lymph node dissection**	
** Yes**	16 (80.0%)
** No**	4 (20.0%)
**Adjuvant treatment**	
** Chemotherapy**	7 (35.0%)
** Immuno plus Chemo**	4 (20.0%)
** Single Immunotherapy**	1 (5.0%)
** Without**	8 (40.0%)

Abbreviations: PC, penile carcinoma; SCC, squamous cell carcinoma.

**Figure 2.**
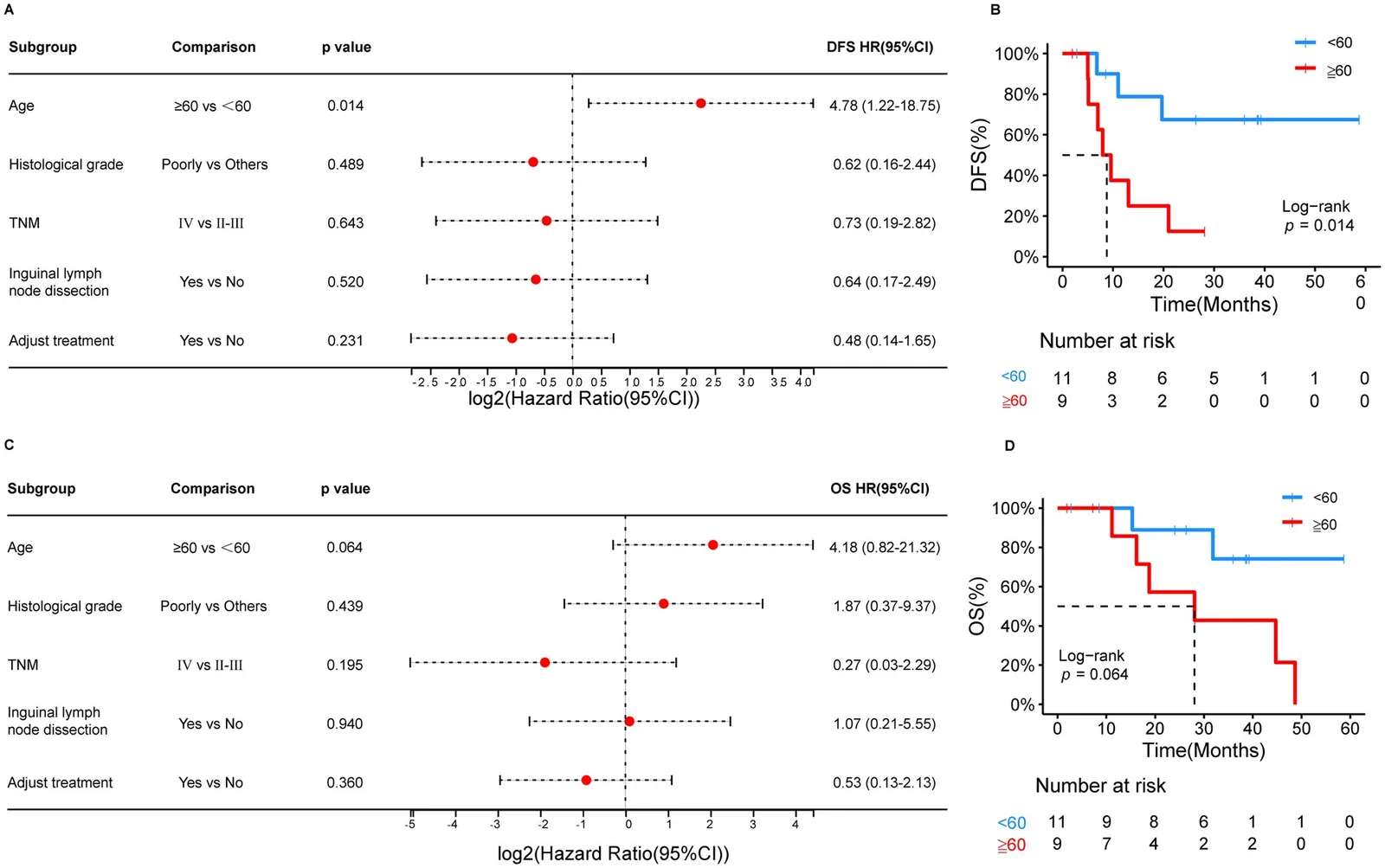
Associations between clinical characteristics and survival outcomes in patients with penile squamous cell carcinoma. (A) Forest plot of univariate Cox regression analysis for disease-free survival (DFS), showing hazard ratios (HRs), 95% confidence intervals (CIs), and *P* values for clinical variables including age, histological grade, TNM stage, inguinal lymph node dissection, and adjuvant therapy. Age ≥60 years was significantly associated with shorter DFS (HR = 4.78, *P* = .014). (B) Kaplan-Meier curve for DFS stratified by age (<60 vs ≥60 years), confirming significantly poorer DFS in older patients (log-rank *P* = .014). (C) Forest plot of univariate Cox regression analysis for overall survival (OS). Although not statistically significant, age ≥60 years showed a trend toward poorer OS (HR = 4.18, *P* = .064). (D) Kaplan-Meier curve for OS stratified by age, demonstrating a similar trend (log-rank *P* = .064).

The median DFS (mDFS) for the cohort was 19.7 months (95% CI: 9.6-not reached, NR), and the median OS (mOS) was 44.7 months (95% CI: 28.1-NR) (Figure S1). Patients aged 60 years or older had significantly shorter DFS than younger patients (8.75 months vs NR, HR = 4.78, 95% CI: 1.22-18.75, *P* = .014; Figure 2A and B) and a nonsignificant trend toward shorter OS (28.1 months vs NR, HR = 4.18, 95% CI: 0.82-21.32, *P* = .064; Figure 2C and D). No other clinical variables, including TNM stage, tumor differentiation, or adjuvant therapy, showed significant associations with DFS or OS (Figure 2A and C).

### Genomic alterations in primary tumors and prognostic associations

**Figure 3.**
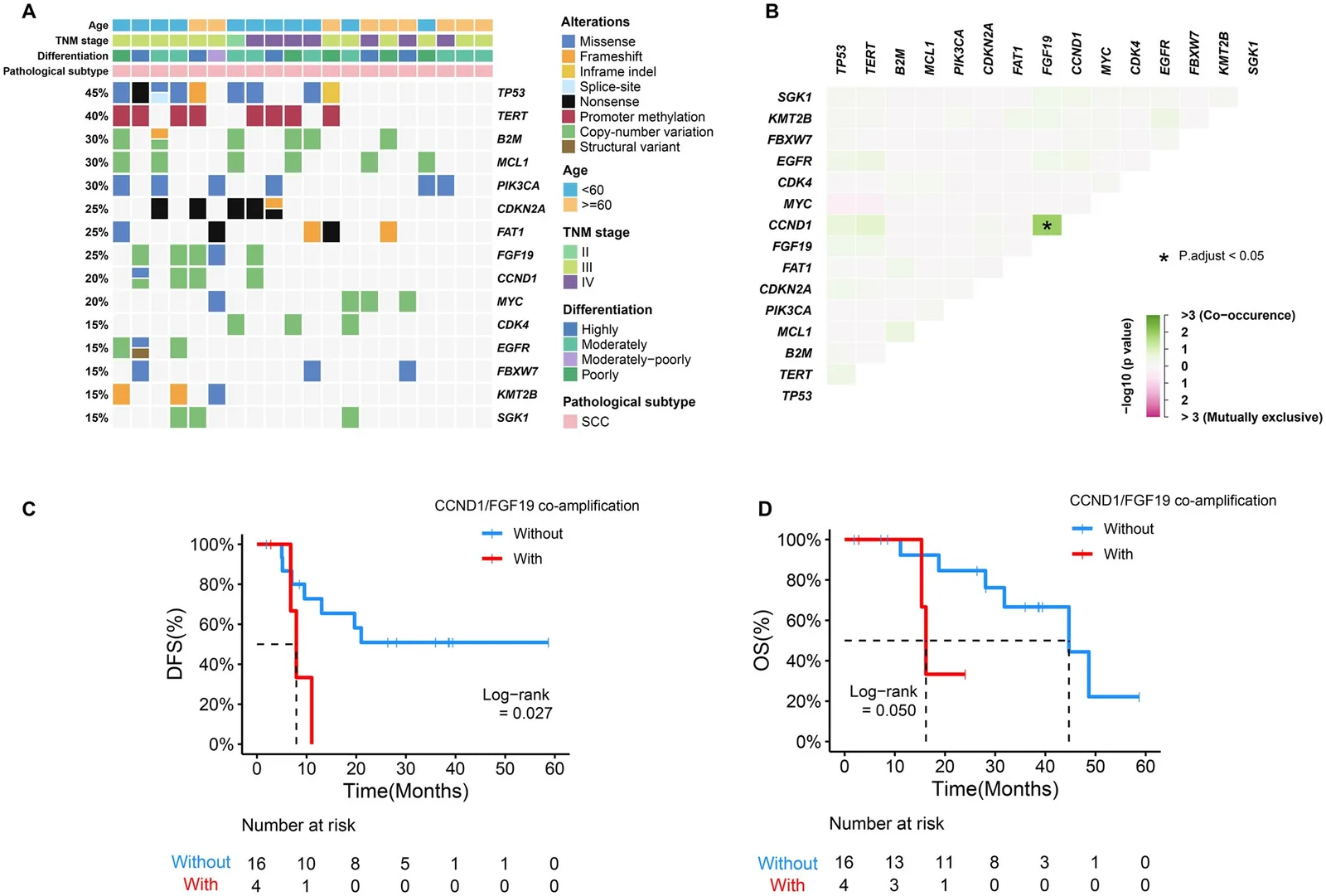
Genomic landscape of primary tumors and prognostic impact of *CCND1/FGF19* co-amplification in penile squamous cell carcinoma. (A) Oncoplot summarizing the most frequently altered genes in primary tumor samples (*n* = 20). Top annotations indicate clinical features including age, histological subtype, TNM stage, and tumor differentiation. Mutation types are indicated by color, including missense, frameshift, CNV, and promoter methylation. (B) Pairwise co-occurrence and mutual exclusivity analysis of genomic alterations. A significant co-occurrence was observed between *CCND1* and *FGF19* alterations (FDR-adjusted *P* < .05, marked by *). (C) Kaplan-Meier curve for disease-free survival (DFS) stratified by the presence or absence of *CCND1/FGF19* co-amplification. Patients with *CCND1/FGF19* co-amplification had significantly shorter DFS (log-rank *P* = .027). (D) Kaplan-Meier curve for overall survival (OS) comparing the same groups, showing a trend toward worse OS in patients with *CCND1/FGF19* co-amplification (log-rank *P* = .050).

All primary tumors harbored at least one gene variant. The most frequently mutated genes included *TP53* (45%), *TERT* (40%), *B2M* (30%), *MCL1* (30%), and *PIK3CA* (30%) (Figure 3A). CNVs were also prominent, particularly in *B2M* (30%), *MCL1* (30%), *FGF19* (20%), and *CCND1* (20%) (Figure 3A). The median TMB was 7.2 mutations per megabase (range: 0-21.6). Co-occurrence analysis revealed a significant association between *CCND1* and *FGF19* alterations (FDR-adjusted *P* < .05) (Figure 3B). Among the five patients with *FGF19* alterations, four harbored *FGF19* amplification, whereas one had an isolated *FGF19* missense mutation without amplification. *CCND1* alterations were detected in four patients, all of whom harbored *CCND1* amplification, with one patient also carried a concurrent *CCND1* missense mutation. Notably, *CCND1* amplification and *FGF19* amplification completely overlapped in the same four patients, who were therefore classified as having *CCND1/FGF19* co-amplification (Figure 3A).

When all five patients with *FGF19* alteration of any type were analyzed together, no significant association was observed with DFS (mDFS, 9.48 vs 21.00 months; HR = 1.973, 95% CI, 0.501-7.765; *P* = .322) or OS (mOS, 16.20 vs 44.70 months; HR = 2.812, 95% CI, 0.452-17.483; *P* = .248; Table S1). By contrast, the four patients with *CCND1/FGF19* co-amplification had shorter DFS than those without co-amplification (mDFS, 7.93 months vs NR; HR = 4.744, 95% CI, 1.040-21.631; *P* = .027; Figure 3C and Table S1) and showed a trend toward shorter OS (mOS, 16.20 vs 44.70 months; HR = 5.913, 95% CI, 0.796-43.896; *P* = .050; Figure 3D and Table S1). Because the gene-specific *CCND1* and *FGF19* amplification occurred in the same four patients, they yielded identical survival estimates. Therefore, an independent prognostic contribution of either gene could not be determined in this cohort. Neither association remained statistically significant after BH correction (*q* = 0.216 for DFS and *q* = 0.402 for OS), and no other gene-level survival association remained significant after multiple-testing correction (Table S1).

Pathway-level analyses showed that patients with T cell receptor (TCR) pathway alterations had longer DFS than patients without these alterations (mDFS: NR vs 7.00 months, HR not estimable, *P* < .001, Figure S2A and Table S2). This association remained statistically significant after BH correction (*q* < 0.001). TCR pathway alterations were also associated with longer OS (mOS: 44.70 vs 17.48 months, HR = 0.21, 95% CI: 0.05-0.94, *P* = .027; Table S2 and Figure S2B), but this association did not remain significant after correction (*q* = 0.487). No other pathway-level survival association remained significant after BH correction. Given the small cohort size and lack of external validation, all gene- and pathway-level survival findings were interpreted as exploratory.

### Genomic heterogeneity between primary and metastatic lesions and its prognostic implications

In the 18 patients with paired primary and metastatic samples, genomic profiles showed substantial overlap, with shared mutations accounting for 59.8% of all alterations. Mutations exclusive to primary tumors represented 18.7%, while those exclusive to metastatic lesions accounted for 21.5% (Figure S3A). Although not statistically significant, *MCL1* amplification tended to be more frequently detected in metastatic lesions (61.1% vs 33.3%, *P* = .112; Figure S3B). Consistent with the overall genomic similarity, pathway enrichment analysis revealed comparable frequencies of alterations across cancer-related pathways such as RTK-RAS, TP53, and Cell Cycle pathways between primary and metastatic lesions (Figure S3D). No differences in the levels of genomic parameters including TMB, CNV, and CIN were observed between primary and metastatic tumors (Figure S3C).

**Figure 4.**
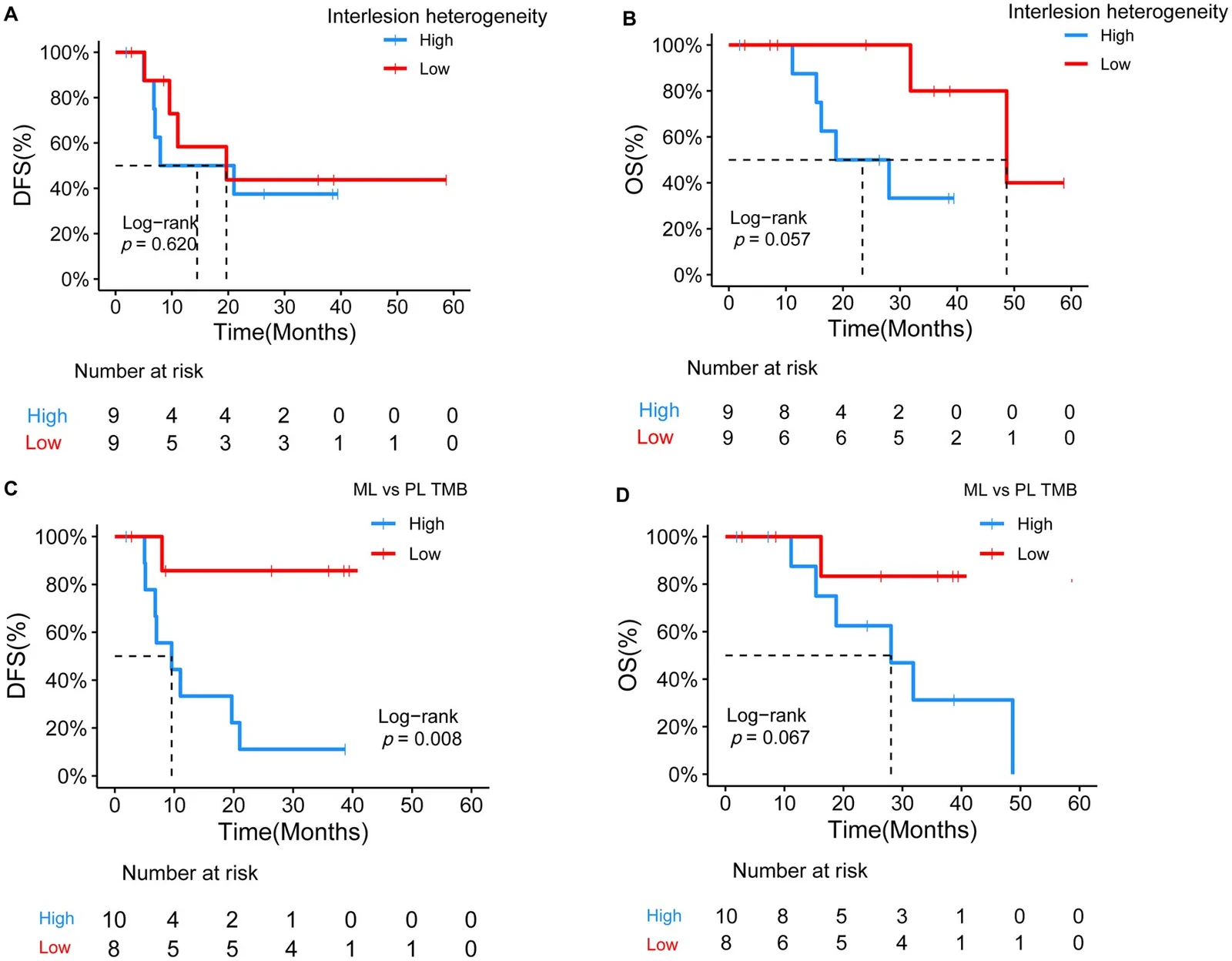
Prognostic impact of interlesion heterogeneity and TMB divergence between metastatic and primary tumors. (A, B) Kaplan-Meier curves for disease-free survival (DFS) (A) and overall survival (OS) (B) stratified by interlesion heterogeneity, defined as the proportion of mutations uniquely present in either the primary or metastatic lesion. Patients with higher interlesion heterogeneity exhibited a trend toward shorter OS (log-rank *P* = .057), although DFS was not significantly affected (*P* = .620). (C, D) Kaplan-Meier curves for DFS (C) and OS (D) based on relative tumor mutation burden (TMB) between metastatic lesions (ML) and matched primary lesions (PL). Patients were classified as “High” if TMB in ML was greater than that in PL, and “Low” if the opposite. A higher TMB in ML relative to PL was significantly associated with shorter DFS (log-rank *P* = .008), and showed a trend toward worse OS (log-rank *P* = .067).

To assess the potential clinical relevance of various genomic correlates, we further analyzed their association with survival. ITH, defined as the proportion of mutations uniquely present in either the primary or metastatic lesion relative to the total number of mutations across both sites, was used to evaluate genomic divergence between paired tumors. Patients with higher ITH exhibited a trend toward worse OS (mOS: 23.42 vs 48.67 months, HR = 0.16, 95% CI: 0.02-1.40, *P* = .057; Figure 4B), although DFS was unaffected (mDFS: 14.47 vs 19.67 months, HR = 0.72, *P* = .620; Figure 4A). Moreover, a higher TMB level in metastatic tumors versus their matched primary was associated with significantly poorer DFS (mDFS: 9.57 vs NR, HR = 0.10, 95% CI: 0.01-0.80, *P* = .008; Figure 4C), and a trend toward reduced OS (HR = 0.17, *P* = .067; Figure 4D). These findings suggest that increased clonal divergence and genomic instability in metastatic sites may reflect more aggressive tumor behavior and contribute to poorer clinical outcomes.

## Discussion

In this study, we performed paired genomic profiling of primary tumors and matched LNMs from Chinese patients with PSCC using targeted NGS. This comprehensive approach enabled the identification of candidate genomic features associated with clinical outcomes and provided insight into tumor evolutionary dynamics. Older age (≥60 years) was significantly associated with shorter DFS and showed a trend toward shorter OS. *CCND1/FGF19* co-amplification was associated with adverse clinical outcomes in this exploratory cohort, representing a potential genomic feature requiring validation in larger studies. Comparisons of matched lesions further identified survival signals related to ITH and metastatic TMB. These findings support the value of paired sequencing in uncovering spatial genomic divergence and highlight its potential to inform prognostic assessment and therapeutic decision-making in this rare malignancy.

Consistent with previous studies, *TP53, TERT,* and *PIK3CA* were among the most frequently mutated genes in primary tumors.^9–13^ We also observed recurrent co-amplification of *CCND1* and its neighboring gene *FGF19*, which was nominal associated with shorter DFS but was not significant after BH correction. *CCND1* encodes cyclin D1, a key regulator of G1/S cell-cycle progression. Prior studies have linked cyclin D1 dysregulation to the biological and clinicopathological heterogeneity of PC. In a recent non-Asian PC cohort, Duarte et al. reported that the absence of cyclin D1 expression was significantly associated with HPV-positive histological subtypes, whereas positive cyclin D1 expression was associated with adverse clinicopathological features, including high-grade tumors, sarcomatoid transformation, and perineural invasion. Patients with cyclin D1 expression also showed numerically shorter DFS, although the survival difference was not statistically significant.^27^ At the genomic level, McDaniel et al. reported recurrent *CCND1* amplification in a non-Asian PSCC cohort and found that *CCND1* amplification was associated with shorter time to progression or PSCC-specific survival.^13^ In a Chinese PC cohort, Chu et al. further demonstrated that HPV DNA and p16 expression were associated with disease-specific survival and other clinicopathological features, highlighting the importance of HPV-related stratification when interpreting cyclin D1/*CCND1*-related findings.^28^ These studies provide relevant biological and clinical context for our observation but not an independent prognostic role for *CCND1* amplification in PSCC.

*CCND1* and *FGF19* are both located within the 11q13 amplicon, a chromosomal region frequently amplified in squamous cell carcinomas and implicated in oncogenic progression.^29–31^ Their complete amplification overlap in our cohort therefore most likely reflects a shared regional copy-number amplification event rather than independent co-selection or direct functional synergy. Moreover, HPV/p16 status was unavailable in our cohort, we could not determine whether the *CCND1/FGF19*-containing 11q13 amplification observed in our study was related to HPV-associated or HPV-independent tumor biology. The finding should therefore be interpreted as an 11q13 amplicon-associated signal rather than evidence for an independent prognostic effect of either gene. Further studies incorporating higher-resolution copy-number mapping, expression profiling, and functional validation are needed to clarify the individual contributions of *CCND1, FGF19,* and other genes within this amplicon to PSCC progression.

*TP53* mutations were not associated with survival in our cohort, but their high prevalence and biological relevance remain notable. *TP53* mutations may promote lymphatic dissemination through loss of p53-mediated checkpoints and altered tumor-stromal interactions.^32^ Previous studies have also suggested that *TP53* alterations can emerge during later tumor evolution and interact with local microenvironments to promote metastasis.^7^^,^^33^^,^^34^

We also observed a favorable prognostic association with mutations in the TCR signaling pathway. Patients harboring TCR pathway alterations had significantly longer DFS, and this association remained statistically significant after BH correction. Although these alterations were also associated with longer OS, the OS association did not remain significant after correction. This signal has rarely been described in PSCC, but interpretation remains limited by the small cohort size, non-estimable Cox effect for DFS, absent HPV stratification, and the lack of an external validation. These mutations may reflect an inflamed tumor microenvironment or enhanced immune surveillance, potentially conferring therapeutic relevance in the context of immunotherapy.^35^^,^^36^ Further studies are warranted to validate this association and to explore whether TCR pathway alterations could predict response to immune checkpoint inhibitors in PSCC.

Most alterations were shared between primary tumors and LNMs, supporting a common clonal origin, but approximately 40% alterations were lesion-specific, indicating continued evolution after dissemination. *MCL1* amplifications were numerically more frequent in metastases, potentially reflecting selection of pro-survival clones during metastatic colonization, although this difference was not statistically significant. At the pathway level, canonical cancer-related pathways such as RTK-RAS, TP53, and cell cycle regulation were altered at similar frequencies in both primary and metastatic tumors, suggesting that the core oncogenic landscape remains largely conserved during progression.

We further evaluated the prognostic significance of spatial heterogeneity between primary and metastatic tumors. Patients with higher ITH showed a trend toward shorter OS, although the difference did not reach statistical significance. This finding is biologically plausible, as increased clonal divergence may reflect enhanced genomic plasticity and the presence of distinct subclones with metastatic potential or treatment resistance.^37^^,^^38^ While DFS did not differ significantly between the groups, the OS trend supports the notion that higher spatial heterogeneity may contribute to long-term adverse outcomes. These results are consistent with findings in other cancers where higher ITH has been linked to worse prognosis.^39^^,^^40^

Additionally, we observed that patients whose metastatic lesions exhibited higher TMB than their matched primary tumors experienced significantly worse DFS and a trend toward inferior OS. This suggests that increased mutational load in metastatic sites may be a marker of genomic instability and aggressive tumor behavior.^41^^,^^42^ Genomic instability has been associated with poor response to therapy and early recurrence in multiple malignancies.^43^^,^^44^ Therefore, dynamic changes in TMB across tumor sites may serve as a valuable indicator of metastatic potential and clinical risk.

The clinical outcomes observed in this cohort should be interpreted within the context of patient selection and treatment characteristics. Although most patients had stage III-IV disease, all patients underwent definitive curative-intent surgery: 80% underwent inguinal lymph node dissection, and 60% received systemic adjuvant therapy. In addition, the stage IV subgroup was heterogeneous, including patients with N3/M0 locoregionally advanced disease and a small number of patients with M1 disease whose known metastatic lesions were completely resected and/or definitively treated. Previous studies have shown that PSCC survival varies substantially with lymph-node burden: limited inguinal involvement may permit favorable long-term survival, whereas extensive nodal disease predicts poorer outcomes.^45^ A population based study identified age, N classification, and the log odds of positive lymph nodes as significant determinants of OS.^46^ The relatively favorable survival in our cohort may therefore reflect surgical selection, inclusion of potentially curable locoregionally advanced or oligometastatic disease, and frequent postoperative therapy; it should not be generalized to all advanced PSCC.

The association between age ≥60 years and worse DFS may also be multifactorial, potentially reflecting comorbidities, reduced physiological reserve, poorer tolerance of surgery or adjuvant therapy, delayed diagnosis, or differences in postoperative recovery and follow-up adherence. Because these factors were not systematically captured, their contributions could not be assessed, and the age association requires validation in larger, more comprehensively annotated cohorts.

This study has several limitations. The small sample size, largely attributable to the rarity of PSCC and the requirement for paired tissues, and the limited number of events reduced statistical power and produced imprecise subgroup estimates, as reflected by several wide CIs. Reliable multivariable adjustment for stage, nodal burden, and adjuvant therapy was not feasible. Clinical heterogeneity and incomplete data on comorbidities, performance status, frailty, diagnostic interval, and treatment adherence also introduced potential residual confounding. Accordingly, the associations involving age, 11q13 co-amplification, TCR pathway alterations, ITH, and metastatic TMB should be considered exploratory.

HPV and p16 status were also unavailable because they were not uniformly documented in historical pathology records and residual FFPE tissue was insufficient for standardized retrospective testing across the entire cohort. Restricting retrospective testing to specimens with adequate remaining tissue would have introduced selection bias and prevented reliable HPV-stratified analyses. We did not infer HPV status from histological appearance or the targeted cancer-gene panel because neither is a validated method of HPV classification. This limitation is important because HPV-associated and HPV-independent PSCC may differ in tumor biology, immune contexture, genomic features, and outcomes, and HPV-independent disease represents a substantial proportion of Chinese PSCC.^28^ Consequently, we could not determine the HPV composition of our cohort or assess whether the association between TCR pathway alterations and favorable outcomes was independent of HPV status or partly reflected HPV-associated immune differences. The absence of HPV stratification also limited our ability to interpret the association between higher TMB in metastatic tumors relative to matched primary tumors and shorter DFS, as well as the nominal adverse survival association observed for *CCND1/FGF19*-containing 11q13 amplification. These findings should therefore be interpreted as exploratory and with appropriate caution. Future prospective studies integrating molecular HPV testing, p16 immunohistochemistry, genomic profiling, and comprehensive immune microenvironment characterization are warranted.

Lastly, the observational design and absence of functional validation prevent causal inference. Prospective studies integrating HPV/p16 assessment, genomic and immune profiling, and experimental validation are warranted.

In conclusion, this study provides an exploratory genomic characterization of primary tumors and matched LNMs in a Chinese PSCC cohort. *CCND1/FGF19*-containing 11q13 amplification was nominally associated with shorter DFS but did not remain significant after BH correction. TCR signaling pathway alterations remained associated with longer DFS after BH correction, although this finding also requires cautious interpretation and external validation. Comparative profiling demonstrated broadly conserved oncogenic pathways between primary and metastatic lesions while revealing spatial genomic divergence and potential prognostic signals related to ITH and metastatic TMB. Collectively, these findings enhance our understanding of PSCC progression and highlight the potential value of NGS for uncovering candidate prognostic markers and generating hypotheses for future translational studies. Larger studies integrating HPV and immune profiling are needed to refine risk stratification and assess clinical relevance.

## Supplementary Material

oyag319_Supplementary_Data

## Data Availability

The datasets generated and analyzed during this current study are available from the corresponding author upon reasonable request.
