# Supplementary material for: Paired genomic profiling of primary tumor and lymph-node metastases identifies candidate prognostic features in penile squamous cell carcinoma
Source: Oncologist. 2026 Aug 27;31(9):oyag319. doi: 10.1093/oncolo/oyag319 (PMC13537821; doi:10.1093/oncolo/oyag319)
Supplement: Supplementary file 1 [file oyag319_supplementary_data.zip › Supplementary Tables.docx]

**Table S1.** Univariate Cox regression analysis of gene-level alterations associated with disease-free survival (DFS) and overall survival (OS) in 20 PC patients.

| Gene | DFS |  |  | OS |  |  |
| --- | --- | --- | --- | --- | --- | --- |
|  | DFS HR (95% CI) | p value | BH-adjusted q value | OS HR (95% CI) | p value | BH-adjusted q value |
| *TP53* | 1.717(0.491~5.999) | 0.392 | 0.570 | 3.559(0.646~19.594) | 0.120 | 0.586 |
| *TERT* | 1.961(0.565~6.807) | 0.280 | 0.532 | 1.921(0.466~7.914) | 0.358 | 0.616 |
| *B2M* | 0.323(0.068~1.537) | 0.135 | 0.433 | 0.887(0.162~4.857) | 0.890 | 0.983 |
| *MCL1* | 0.418(0.089~1.973) | 0.256 | 0.532 | 0.954(0.174~5.229) | 0.957 | 0.983 |
| *PIK3CA* | 0.36(0.076~1.702) | 0.179 | 0.476 | 0.399(0.078~2.048) | 0.256 | 0.586 |
| *CDKN2A* | 0.374(0.047~2.964) | 0.333 | 0.532 | 0.399(0.047~3.386) | 0.385 | 0.616 |
| *FAT1* | 0.896(0.228~3.51) | 0.874 | 0.932 | 2.315(0.463~11.581) | 0.293 | 0.586 |
| Any *FGF19* alteration | 1.973(0.501~7.765) | 0.322 | 0.532 | 2.812(0.452~17.483) | 0.248 | 0.586 |
| *FGF19* amplification | 4.744(1.04~21.631) | 0.027 | 0.216 | 5.913(0.796~43.896) | 0.050 | 0.402 |
| *CCND1* amplification | 4.744(1.04~21.631) | 0.027 | 0.216 | 5.913(0.796~43.896) | 0.050 | 0.402 |
| *MYC* | 0.84(0.178~3.965) | 0.826 | 0.932 | 0.73(0.144~3.693) | 0.703 | 0.983 |
| *CDK4* | NE(0~Inf) | 0.066 | 0.353 | NE(0~Inf) | 0.201 | 0.586 |
| *EGFR* | 2.822(0.699~11.382) | 0.128 | 0.433 | 2.837(0.512~15.705) | 0.212 | 0.586 |
| *FBXW7* | 1.542(0.324~7.324) | 0.583 | 0.778 | 1.024(0.195~5.366) | 0.978 | 0.983 |
| *KMT2B* | 0.991(0.209~4.701) | 0.991 | 0.991 | 0.836(0.097~7.201) | 0.870 | 0.983 |
| *SGK1* | 1.288(0.271~6.119) | 0.750 | 0.923 | 1.024(0.118~8.885) | 0.983 | 0.983 |

***Notes:*** HRs and 95% CIs were estimated using univariate Cox proportional hazards models, and P values were derived from two-sided log-rank tests. The Benjamini-Hochberg procedure was applied separately to the DFS and OS analyses across all gene-level alteration variables included in this table. A BH-adjusted q value<0.05 was considered statistically significant after correction. Any *FGF19* alteration included four amplifications and one isolated missense mutation without amplification. *FGF19* amplification and *CCND1* amplification occurred in the same four patients, representing *CCND1/FGF19* co-amplification, and therefore yielded identical survival estimates.

Abbreviations: BH, Benjamini-Hochberg; CI, confidence interval; DFS, disease-free survival; HR, hazard ratio; NE, not estimable; OS, overall survival; PSCC, penile squamous cell carcinoma.

**Table S2.** Univariate Cox regression analysis of pathway-level alterations associated with disease-free survival (DFS) and overall survival (OS) in 20 PC patients.

| Pathway | DFS |  |  | OS |  |  |
| --- | --- | --- | --- | --- | --- | --- |
|  | DFS HR (95% CI) | P value | BH-adjusted q value | OS HR (95% CI) | P value | BH-adjusted q value |
| Immune-related pathways | | | | | | |
| Homologous recombination | 1.151(0.145~9.151) | 0.894 | 0.906 | 0.633(0.068~5.938) | 0.687 | 0.888 |
| Nucleotide excision repair | NE (0~Inf) | 0.117 | 0.548 | 0(0~Inf) | 0.121 | 0.543 |
| TNF family members and receptors | 1.429(0.294~6.943) | 0.657 | 0.844 | 1.339(0.156~11.503) | 0.789 | 0.888 |
| Cytokines | 1.874(0.526~6.668) | 0.325 | 0.705 | 4.093(0.674~24.859) | 0.098 | 0.543 |
| Antigen processing and presentation | 0.547(0.14~2.134) | 0.378 | 0.705 | 1.698(0.342~8.428) | 0.512 | 0.888 |
| Antimicrobials | 0.713(0.201~2.533) | 0.599 | 0.829 | 0.769(0.182~3.25) | 0.720 | 0.888 |
| Cytokine receptors | 1.122(0.314~4.016) | 0.859 | 0.906 | 0.935(0.222~3.939) | 0.927 | 0.976 |
| Natural killer cell cytotoxicity | 0.402(0.113~1.431) | 0.146 | 0.548 | 0.713(0.177~2.87) | 0.632 | 0.888 |
| B-cell receptor signaling | 0.412(0.118~1.441) | 0.152 | 0.548 | 0.978(0.231~4.131) | 0.976 | 0.976 |
| T-cell receptor signaling | NE(0~Inf) | <0.001 | <0.001 | 0.206(0.046~0.936) | 0.027 | 0.487 |
| Tumor-realted pathways | | | | | | |
| RTK-RAS | 1.543(0.325~7.312) | 0.582 | 0.829 | 1.525(0.303~7.679) | 0.606 | 0.888 |
| TP53 | 1.717(0.491~5.999) | 0.392 | 0.705 | 3.559(0.646~19.594) | 0.120 | 0.543 |
| PI3K | 0.31(0.079~1.216) | 0.077 | 0.548 | 0.47(0.11~2.016) | 0.299 | 0.792 |
| Cell cycle | 0.502(0.13~1.947) | 0.310 | 0.705 | 0.488(0.098~2.444) | 0.373 | 0.839 |
| NOTCH | 0.625(0.161~2.419) | 0.492 | 0.805 | 0.426(0.079~2.293) | 0.308 | 0.792 |
| MYC | 0.477(0.101~2.251) | 0.339 | 0.705 | 0.553(0.111~2.763) | 0.464 | 0.888 |
| HIPPO | 1.08(0.301~3.87) | 0.906 | 0.906 | 2.197(0.488~9.899) | 0.293 | 0.792 |
| WNT | 0.873(0.185~4.119) | 0.863 | 0.906 | 0.798(0.157~4.063) | 0.785 | 0.888 |

***Notes:*** HRs and 95% CIs were estimated using univariate Cox proportional hazards models, and unadjusted P values were derived from two-sided log-rank tests. The Benjamini–Hochberg procedure was applied separately to the DFS and OS analyses across all 18 pathway-level variables included in this table. A BH-adjusted q value <0.05 was considered statistically significant after correction. The exact unadjusted P value and BH-adjusted q value for the association between T-cell receptor signaling pathway alterations and DFS were 4.0×10⁻⁶ and 7.2×10⁻⁵, respectively.

Abbreviations: BH, Benjamini–Hochberg; CI, confidence interval; DFS, disease-free survival; HR, hazard ratio; NE, not estimable; OS, overall survival; PSCC, penile squamous cell carcinoma; RTK, receptor tyrosine kinase; TCR, T-cell receptor; TNF, tumor necrosis factor.

**Table S3.** Clinical characteristics and treatment of the seven patients with stage IV penile squamous cell carcinoma.

| Case No. | Age, years | Differentiation | TNM classification | Inguinal lymph node dissection | Adjuvant treatment |
| --- | --- | --- | --- | --- | --- |
| 1 | 79 | Well differentiated | T1N3M0 | No | None |
| 2 | 52 | Moderately differentiated | T3N3MX | Yes | Chemotherapy |
| 3 | 73 | Moderately differentiated | T4N2M1 | Yes | Chemotherapy |
| 4 | 57 | Well differentiated | T2N1M1 | Yes | Chemotherapy |
| 5 | 67 | Well differentiated | T2N1M1 | Yes | Single Immunotherapy |
| 6 | 47 | Moderately differentiated | T2N3M0 | Yes | None |
| 7 | 40 | Poorly differentiated | T1N3M0 | Yes | Immuno plus Chemo |
