# Supplementary material for: Paired genomic profiling of primary tumor and lymph-node metastases identifies candidate prognostic features in penile squamous cell carcinoma
Source: Oncologist. 2026 Aug 27;31(9):oyag319. doi: 10.1093/oncolo/oyag319 (PMC13537821; doi:10.1093/oncolo/oyag319)
Supplement: Supplementary file 1 [file oyag319_supplementary_data.zip › Figure s3.pdf]

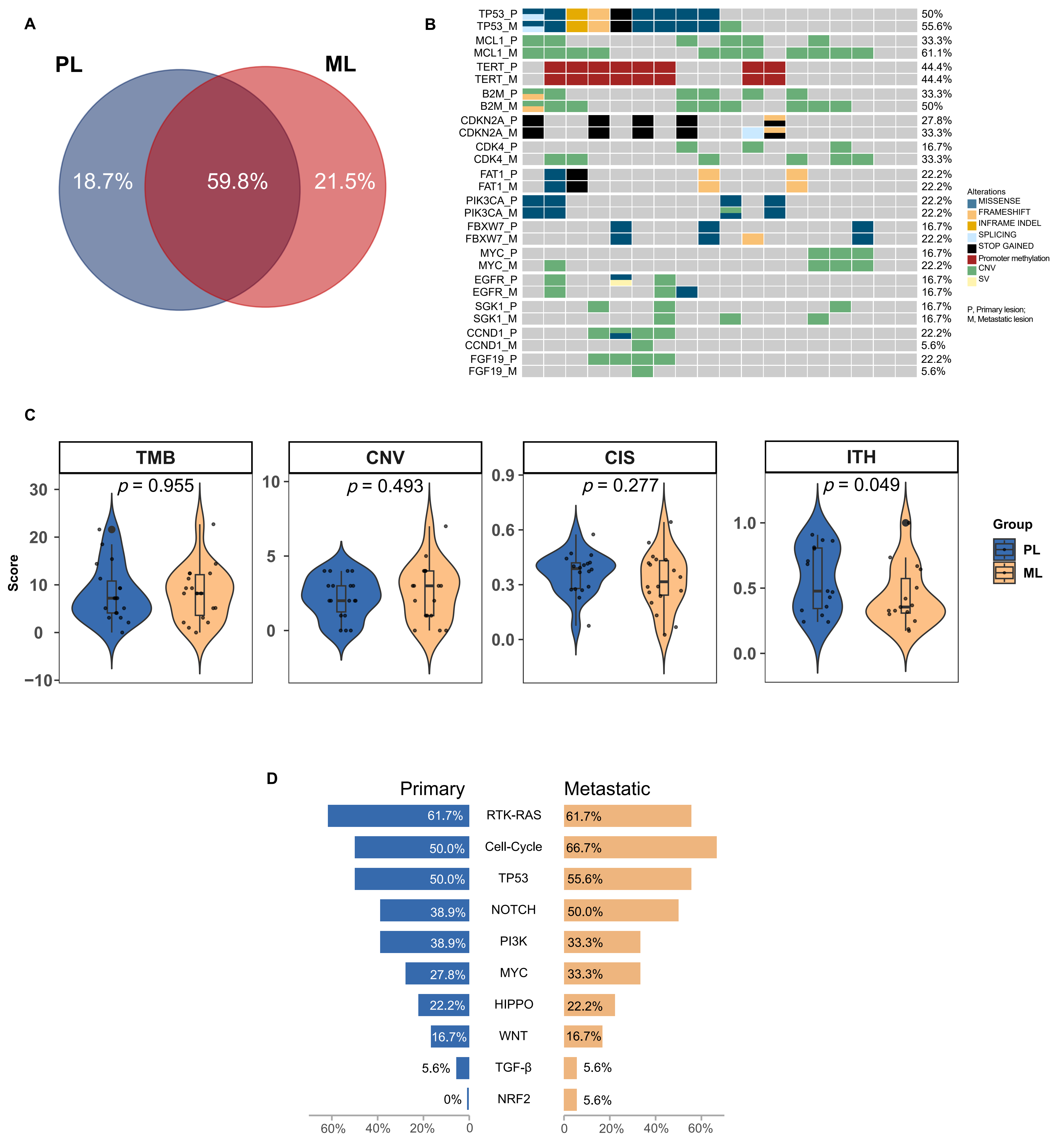

A
PL
ML
18.7%
59.8%
21.5%
B
TP53_P 50%
TP53_M 55.6%
MCL1_P 33.3%
MCL1_M 61.1%
TERT_P 44.4%
TERT_M 44.4%
B2M_P 33.3%
B2M_M 50%
CDKN2A_P 27.8%
CDKN2A_M 33.3%
CDK4_P 16.7%
CDK4_M 33.3%
FAT1_P 22.2%
FAT1_M 22.2%
PIK3CA_P 22.2%
PIK3CA_M 22.2%
FBXW7_P 16.7%
FBXW7_M 22.2%
MYC_P 16.7%
MYC_M 22.2%
EGFR_P 16.7%
EGFR_M 16.7%
SGK1_P 16.7%
SGK1_M 16.7%
CCND1_P 22.2%
CCND1_M 5.6%
FGF19_P 22.2%
FGF19_M 5.6%
Alterations
MISSENSE
FRAMESHIFT
INFRAME INDEL
SPLICING
STOP GAINED
Promoter methylation
CNV
SV
P, Primary lesion;
M, Metastatic lesion
C
TMB p = 0.955
CNV p = 0.493
CIS p = 0.277
ITH p = 0.049
Score
Group
PL
ML
D
Primary
Metastatic
RTK-RAS 61.7% 61.7%
Cell-Cycle 50.0% 66.7%
TP53 50.0% 55.6%
NOTCH 38.9% 50.0%
PI3K 38.9% 33.3%
MYC 27.8% 33.3%
HIPPO 22.2% 22.2%
WNT 16.7% 16.7%
TGF-β 5.6% 5.6%
NRF2 0% 5.6%
60% 40% 20% 0
0 20% 40% 60%
