# Supplementary material for: Paired genomic profiling of primary tumor and lymph-node metastases identifies candidate prognostic features in penile squamous cell carcinoma
Source: Oncologist. 2026 Aug 27;31(9):oyag319. doi: 10.1093/oncolo/oyag319 (PMC13537821; doi:10.1093/oncolo/oyag319)
Supplement: Supplementary file 1 [file oyag319_supplementary_data.zip › Supplementary Figure Legends.docx]

**Figure S1. Kaplan-Meier estimates of disease-free and overall survival in the entire cohort.**

(A) Kaplan-Meier curve showing disease-free survival (DFS) for all patients (n = 20). The median DFS was 19.7 months (95% CI: 9.6–not reached).

(B) Kaplan-Meier curve showing overall survival (OS) for all patients (n = 20). The median OS was 44.7 months (95% CI: 28.1–not reached).

**Figure S2. Prognostic significance of T cell receptor (TCR) pathway alterations in patients with penile squamous cell carcinoma.**

(A) Kaplan-Meier curve for disease-free survival (DFS) in patients with TCR pathway alterations (abnormal, n = 12) versus those without (normal, n = 8). Patients with TCR pathway alterations exhibited significantly improved DFS (log-rank p < 0.0001).

(B) Kaplan-Meier curve for overall survival (OS) in the same groups. TCR pathway alterations were also associated with prolonged OS (log-rank p = 0.027).

**Figure S3. Comparative genomic profiling between primary tumors and matched lymph node metastases in penile squamous cell carcinoma.**

(A) Venn diagram showing the proportion of shared and lesion-specific mutations in primary lesions (PLs) and metastatic lesions (MLs) among the 18 patients with paired sequencing data.

(B) Heatmap showing genomic alterations in selected cancer-related genes in the paired primary and metastatic samples from the same 18 patients. Rows ending in “_P” represent alterations detected in primary lesions, whereas rows ending in “_M” represent alterations detected in the corresponding matched metastatic lesions. Each column represents the same patient across the paired lesion types. The percentages shown on the right indicate the proportion of patients harboring an alteration in the corresponding gene and lesion type. Alteration categories are indicated by color as defined in the figure key.

(C) Violin plots comparing tumor mutation burden (TMB), copy number variation (CNV) scores, chromosomal instability (CIN), and intralesional heterogeneity (ITH) between PL and ML. P values were calculated using the two-sided Wilcoxon signed-rank test. ITH was significantly higher in PLs than in matched MLs (p = 0.049).

(D) Frequency of pathway-level alterations across canonical oncogenic pathways in primary versus metastatic tumors. The most frequently altered pathways were RTK-RAS, Cell Cycle, and TP53 in both compartments.
